# Experimental evidence of selective inattention in reputation-based cooperation

**DOI:** 10.1038/s41598-018-33147-x

**Published:** 2018-10-04

**Authors:** Isamu Okada, Hitoshi Yamamoto, Yoshiki Sato, Satoshi Uchida, Tatsuya Sasaki

**Affiliations:** 1 Department of Business Administration, Soka University, Tangi 1-236, Hachioji, Tokyo 192-8577 Japan; 2 Department of Business Administration, Rissho University, Osaki 4-2-16, Shinagawa, Tokyo 141-8602 Japan; 3 The United Graduate School of Education, Tokyo Gakugei University, Nukuikita 4-1-1, Koganei, Tokyo 184-8501 Japan; 4 Info Screw Inc., Sakaecho 13-6, Itabashi, Tokyo 173-0015 Japan; 5 Research Center for Ethi-Culture Studies, RINRI Institute, Kioicho 4-5, Chiyoda, Tokyo 102-8561 Japan; 6 Faculty of Mathematics, University of Vienna, Oskar-Morgenstern-Platz 1, 1090 Vienna, Austria; 7 F-power Inc., Roppongi 1-8-7-2F, Minato, Tokyo 106-0032 Japan

## Abstract

Reputation-based cooperation is often observed in modern society. People gain several types of information by assessing others. Among these, the most important information is the actions of people and those of their recipients. However, almost all studies assume that people consider all of the information they receive. This assumption is extreme, and people engaging in reputation-based cooperation may not pay attention to some information, i.e., they may display selective inattention. We demonstrate that subjects’ decision-making in relation to cooperative action depends on the content of the information they receive about their recipients. Our results show that subjects either consider or ignore information depending on the content of that information. When their recipients had cooperated previously, subjects cooperated without considering the information they received. When the recipients had played before with those who had bad reputations, subjects did not use that information, regardless of whether it was disclosed proactively. In other cases, subjects considered information on both the previous actions of recipients and those of the recipients’ own recipients. We found that subjects did not always use the information to make decisions, although they willingly received information about their recipients. This supports the proposition that selective inattention occurs in reputation-based cooperation.

## Introduction

The concept of cooperation^1,2^ remains a mystery, even though many disciplines in the social sciences have devoted considerable attention to this topic. Numerous studies on the evolution of cooperation have addressed this issue, and have considered many mechanisms including the effect of imitating of a more successful player^3^. If an opportunity for contribution occurs repeatedly among a group of individuals, direct reciprocity is an acceptable solution to this mystery^2^. However, when repetition does not occur, this type of cooperative mechanism fails. Indirect reciprocity^4,5^ is a more significant solution as it requires neither kinship^6^ nor a network structure^7^ and is suitable for analysing large-scale and highly flexible systems that are often observed in modern society. In these systems, players consider whether to cooperate after considering information about other players. Hence, indirect reciprocity requires reputation while direct reciprocity requires repetition^8^. We can observe reputation-based cooperation in many online services including Amazon, eBay, and CouchSurfing. In this study, reputation is defined as the image (either good or bad) of an individual or supporter playing an indirect reciprocity game in which they help those with good images and do not help those with bad images^9^.

Reputation-based cooperation focuses on assessment rules used by players^10,11^. Many theoretical^12–14^ and empirical^9,15–17^ studies have revealed that players obtain several types of information they can use to assess other players. Of these, the most important types relate to the previous actions of players and those of their recipients (hereafter “what data” and “whom data”, respectively). In the image-scoring norm, which is an assessment rule proposed by Nowak and Sigmund^18^, cooperative players obtain a good reputation, while defective players develop a bad reputation. Thus, in the norm, whether players cooperated or defected (what data) is important information. In the simple-standing norm^19^, which is more tolerant than the image-scoring norm, players will develop a good reputation when they not only cooperate with others but also defect against those who have a bad reputation. Thus, in the norm, whom data as well as what data is considered when assessing players.

Despite numerous studies on assessment rules, it remains unclear what type of rules are suitable for maintaining and promoting cooperative regimes. Theoretically, image-scoring cannot justify any punitive defection because such a defection downgrades one’s reputation. Thus, it does not maintain cooperative regimes^8,20^. This theory suggests that both what data and whom data are needed for stable cooperation. However, according to the results of an experiment conducted by Millinski^16^, people do not like to process complex information when they can assess others on the basis of less complex information, i.e., what data. Swakman *et al*. (2016) raised an objection to their findings^10^, stating that even if there is a cost associated with obtaining information, people make decisions on the basis of both what data and whom data.

In searching for a new solution regarding the assessment rules, selective inattention^21^ has been considered, which is a psychological phenomenon that has been widely observed in many fields^22,23^ where refuses to notice or acknowledge details they do not wish to know. On the one hand, most studies on reputation-based cooperation have dealt with assessment rules that share the feature that assessors thoroughly assess each play. On the other hand, some studies^24,25^ have explored a new assessment rule, termed ‘staying’, that involves selective inattention. In staying, the assessors do not always assess each play. While this is pioneering work, it has not been validated by empirical evidence.

In this study, we demonstrate that selective inattention occurs in reputation-based cooperation. When participants in a social dilemma game make a decision on whether to cooperate with their recipient players, their decision-making depends on the content of the information they receive regarding their recipients. If the recipients previously played with players who had bad reputations, the information received about their recipients does not significantly influence participants’ decision-making. If other players had good reputations, the participants’ decision-making depends on their recipients’ previous actions.

## Results

**Figure 1:**
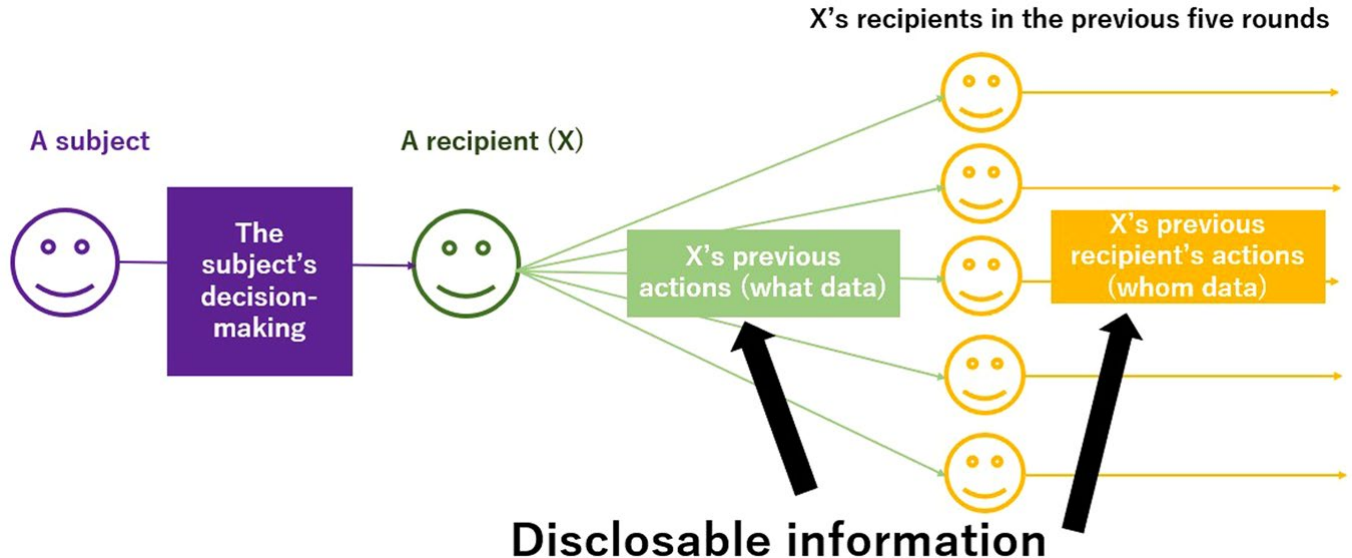
Disclosable information subjects can receive. This illustration shows the information disclosed to subjects in an ‘indirect helping game’ before they decide on their actions (cooperation or defection) in each round. Every subject meets a recipient, called X, who is assigned by the system in each round. The information about X falls into two categories. First, X’s actions (what data) in the previous five rounds are disclosable. For example, if what data (X’s previous action) in the third-last round is disclosed, either ‘C’ (cooperation) or ‘D’ (defection) is shown. Second, X’s recipients’ actions (whom data) in the previous five rounds are also disclosable. For example, if whom data (about X’s recipient) in the third-last round is disclosed, an integer between 0 and 5 is shown. The integer represents how many times X’s recipient cooperated in the five rounds preceding that round (the number of ‘C’ values), in this case, the five rounds from the fourth-last round to the eighth-last round. Therefore, the total number of pieces of disclosable information about X is ten (five items of what data and five items of whom data).

We conducted an experiment using ECoSoS, a self-developed computer-based system, with 152 subjects who accessed the system using a Web browser (see Methods). Our experiment focused on behavioural differences relating to information disclosure. Subjects played repeated rounds of an ‘indirect helping game’. Using the setup shown in Fig. 1, in each round, a subject could receive information about the actions of a recipient and those of the recipient’s own recipients in the previous five rounds at no cost before the subject decided whether to cooperate with the recipient. Note that all data (five instances of what data and whom data) were made available to subjects before deciding. Subjects could receive at most four items of either what or whom data by pushing the disclosure button on the information disclosure screen.

### Information disclosure behaviour

**Figure 2:**
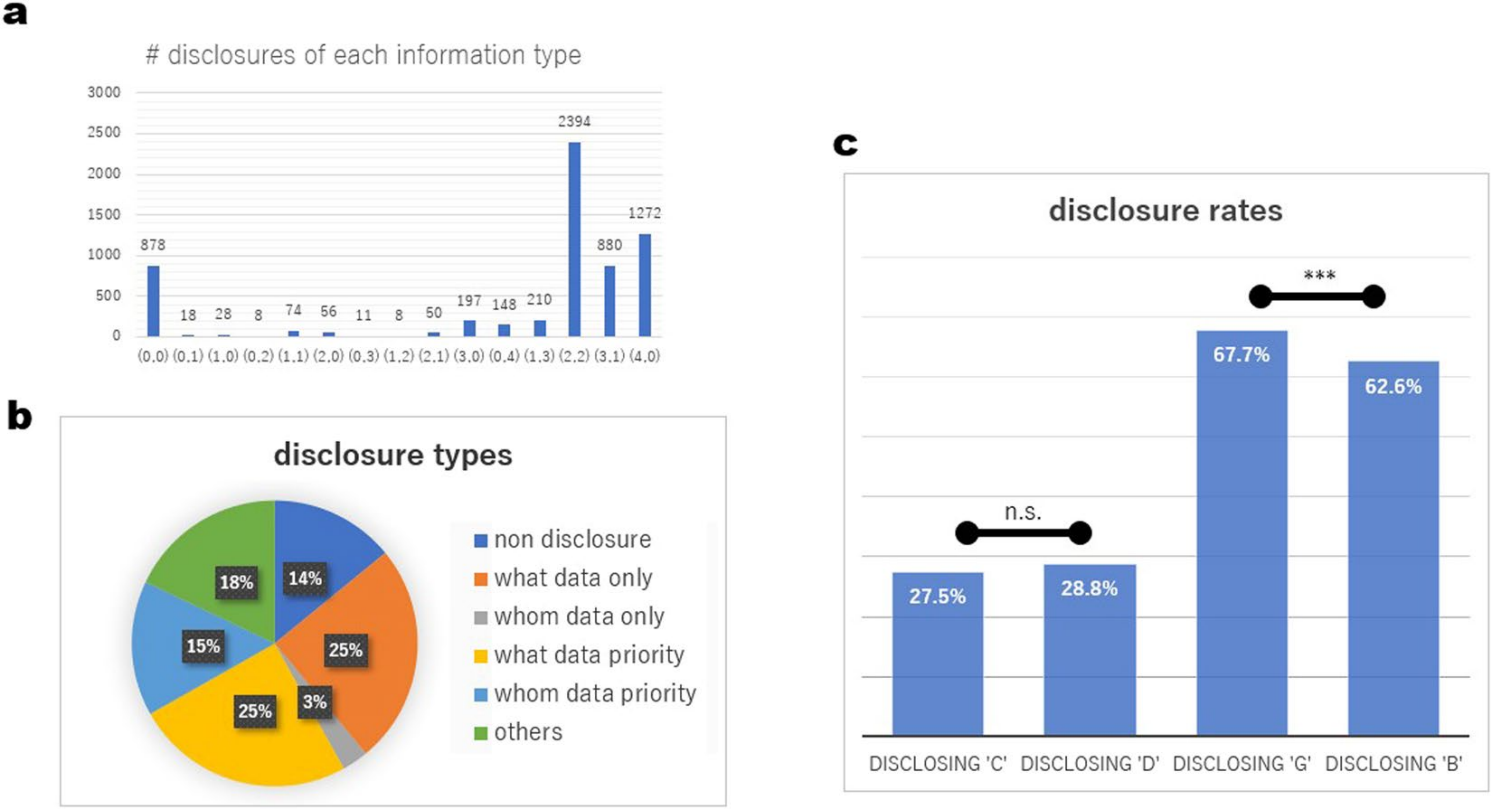
Information disclosure behaviour. The graphs show the disclosure behaviour of all subjects (N = 152) except for the first ten rounds and the 52nd and later rounds (# = 6,232). (**a**) The number of disclosures in the case of (x, y) in each round where x and y represent the number of disclosures of what data and whom data, respectively. (**b**) The number of disclosure types. These were counted for each subject in each round. To define what data priority, we introduce the concept of a data pair and a what-prior pair. A data pair consists of what data and its corresponding whom data a data pair. A what-prior pair is a data pair in which its what data was disclosed before its whom data (including the case where the whom data was not disclosed). If all five data pairs in a round are what-prior pairs, this set of pairs is said to have what data priority. Whom data priority is defined as having all five data pairs in a round as whom-prior pairs (whom data is disclosed before the what data). (**c**) The disclosure rates of pairs (what data and whom data in the same round) and the Fisher’s exact test values for all pairs. For example, “DISCLOSING ‘C’” is the disclosure rate of whom data when the previously disclosed what data for the pairs were ‘C’. The Fisher’s exact test values show that the content of what data has no statistically significant effect on disclosed behaviour in relation to whom data, while the content of whom data has a statistically significant effect on disclosed behaviour in relation to what data. Statistics are shown in Table S1 in Supplementary Information.

Our analysis of the results of the experiment shows that information disclosure behaviour exhibits several features (Fig. 2). First, the top four situations in terms of the number of disclosures are as follows: two items of what data and two items of whom data (38%), four items of what data (20%), three items of what data and one item of whom data (14%) and no items (14%) (Fig. 2a). To determine the relationship between disclosed what data and disclosed whom data, Fig. 2b shows the non-negligible number of cases of whom data priority (15%; defined in the caption of Fig. 2) as well as those of what data priority (25%), while those in which only what data are disclosed (25%) are the most frequent.

Most studies on reputation-based cooperation refer to what data and whom data as first-order and second-order information, respectively^4^. These terms imply that second-order information is only disclosed after first-order information has been disclosed. However, our experiment showed that players observe what data and whom data independently. Our results show a case in which first-order information is disclosed in accordance with the content of the second-order information that was previously disclosed.

The second feature of information disclosure behaviour is that it depends on the content of previous disclosures (Fig. 2c). In this study, the labels ‘G’ (good) and ‘B’ (bad) correspond to whom data values of ‘3 or more’ and ‘2 or less’ ‘C’ (cooperation) actions, respectively, in the previous five rounds. The Fisher’s exact test results for all subjects show that the disclosure rate for what data when whom data that was previously disclosed was ‘G’ is significantly higher than the disclosure for that when whom data that was previously disclosed was ‘B’ (P value = 0.0007029, one-tailed test). In contrast, there was no significant difference in the disclosure rate of whom data when what data that was previously disclosed was either ‘C’ or ‘D’ (P = 0.1384).

Theoretically, there is no reason for players not to receive what data even though the whom data in the pair had previously been disclosed. Thus, as shown in Fig. 2c, more than 60% of what data were disclosed when the whom data in the pair had previously been disclosed, while less than 30% of whom data were disclosed when the what data in the pair had previously been disclosed. Fisher’s exact test values in relation to the cases of ‘G’ and those of ‘B’ suggest that players tended not to care about information when whom data were ‘B’. To determine if this evidence of selective inattention, we needed to perform a statistical analysis of cooperative actions.

### Cooperation and information disclosure

We tested whether information disclosure behaviour influences decision-making. To do this, we categorized each round of each subject into seven models and applied those with logistic generalized linear mixed models, as shown in Table 1. Model 1 (with no disclosure) shows that decisions regarding cooperation are positively influenced by three factors (subjects’ current total earnings, the decisions by subjects in the previous two rounds and whether they previously received help as a recipient) and negatively influenced by one factor (the round number), as found by many previous studies^15,26^.

**Table 1:** Determinants of cooperation.

Model	(1)	(2)	(3)	(4)	(5)	(6)	(7)
what data		1.017***		−0.132	1.282***	1.395***	0.265
whom data			0.219	0.073	−0.287***	−0.167	−0.133
recent decision	1.671***	0.695***	1.198***	0.697***	0.475***	1.206***	0.953***
round	−0.029**	−0.013*	−0.019	−0.015	−0.028**	−0.043**	0.021
current total earnings	2.834***	1.607***	0.872	1.665**	3.743***	1.959*	1.020
decision received	0.883***	0.391**	0.211	0.966***	0.510*	0.705*	0.439
intercept	−2.174**	−1.139***	−0.725	−0.696+	−1.084**	−1.182*	−1.200*
N	878	1,553	185	535	625	296	254

Each column presents estimates of the fit of a logistic generalized linear mixed model to decisions to cooperate (see Supplementary Information). In all models, we controlled the round number, subjects’ current total earnings divided by 10,000, the decisions by subjects in the previous two rounds (the number of ‘C’ between 0 and 2), and previously received help as a recipient in the previous round (‘C’ and ‘D’ are coded as 1 and 0, respectively). Model (2) and Models (4) to (7) consider the average number of items of what data disclosed while Models (3) to (7) consider those of whom data disclosed. Data from the first ten rounds and beyond the 51st round were excluded from the analysis. Data for Model (1) include only those decisions with no disclosure. Models (2) and (3) include those decisions in which only what data and only whom data, respectively, were disclosed. Models (4) and (5) include those decisions in which all pairs are what-prior data and the what data are ‘C’ only and ‘D’ only, respectively. Models (6) and (7) include those decisions in which all pairs are whom-prior data and the whom data are ‘G’ only and ‘B’ only, respectively. The remaining data (N = 1,906) were excluded from the analysis. P-values: *** < 0.1% < ** < 1% < * < 5% < + < 10%, where the P-value is defined as the minimum level of significance that is needed to reject the null hypothesis in relation to each variable.

Our experiment supports the proposition that what data and whom data play completely different roles in terms of cooperative behaviour. Model 2 shows that subjects reacted to what data in a strongly reciprocal way; they cooperated with others who had cooperated previously, consistent with the findings of Swakman *et al*.^10^. Such ‘indirect reciprocity’ is widely observed and repeatedly studied. In contrast, Model 3 shows that whom data had little influence on decision-making when subjects received only whom data. However, this is a rare situation because those included in Model 3 only comprise 3.0% of all cases, while the number comprising Model 2 is about eight times greater (25.0%). This is theoretically true because the whom data have no relationship with the recipients themselves, and thus disclosing whom data without disclosing what data is entirely irrational. Subjects may be confused by the situations. Model 3 has no significant factors except for previous decisions by the subjects themselves.

We tested the aggregate effects of what data and whom data in terms of decision-making regarding cooperation by comparing Models 4 to 7. First, we considered the cases of what-prior pairs (Models 4 and 5). When the what data of what-prior pairs were ‘D’, the whom data of the pairs (either ‘G’ or ‘B’) had a strong effect on decision-making (Model 5). However, the results of Model 4 showed that the content of whom data had no effect on decision-making when the what data of what-prior pairs were ‘C’. This is inconsistent with Swakman *et al*.’s findings (Model 3 in their paper), and thus we need to conduct further experiments.

Comparing Models 4 and 5, attention by the players depends on the content of what-prior data. On the one hand, if the what data are ‘C’, neither what data nor whom data have a significant influence on their decision-making. Helping others who cooperated in the previous round corresponds to the mechanism of ‘indirect reciprocity,’ whereby the player helps the recipient because they helped someone else. On the other hand, if the what data are ‘D’, both what data and whom data have a significant influence on their decision-making. Subjects check whether or not the recipients’ previous defections are justified. ‘Justified defection’^17^ has been repeatedly considered by many theoretical and empirical papers.

Second, we consider the case of whom-prior pairs. Unprecedented insights are revealed by a comparison of Models 6 and 7. When the whom data in whom-prior pairs were ‘G’, the what data of the pairs (either ‘C’ or ‘D’) had a strong effect on decision-making (Model 6). If recipients cooperated with good players in the previous round, subjects chose cooperation. This ‘prosociality’^27,28^ or ‘prosocial chain’ is regarded as an extended version of ‘indirect reciprocity’, that is, a player helps a recipient because they helped good people, or a player refuses to help a recipient because they refused to help good people.

In contrast to Model 6, Model 7 shows that when the whom data in whom-prior pairs were ‘B’, the what data in the pairs had no significant influence on reputation-based cooperation. Even if recipients had played with bad players in the previous round, subjects did not care about this information. This is evidence of selective inattention playing a role in reputation-based cooperation.

### Selective inattention

In most studies on the norms of reputation-based cooperation, it has been debatable how people assess donors’ actions when the donors play with those who have bad reputations. For example, Kandori (1992)^29,30^ applied a rigorous rule, later termed stern-judging, whereby cooperation with bad players is assessed as bad and defection to bad players is assessed as good. Sugden^19^ proposed a more tolerant rule, later termed simple-standing, as mentioned earlier. These rules have been compared numerous times, both theoretically^12,31^ and empirically^16^. The results of our study suggest that the possibility of selective inattention should be a main focus. The data presented in Table 1 suggest that subjects intentionally ignore information regarding reputations in specific situations. In particular, Model 7 indicates the existence of selective inattention, whereby subjects do not consider the previous actions of those who have bad reputations. This empirical fact is entirely consistent with the staying norm^24,25,32^ because this norm ignores recipients’ actions and maintains their reputations even when their previous recipients had bad reputations.

**Figure 3:**
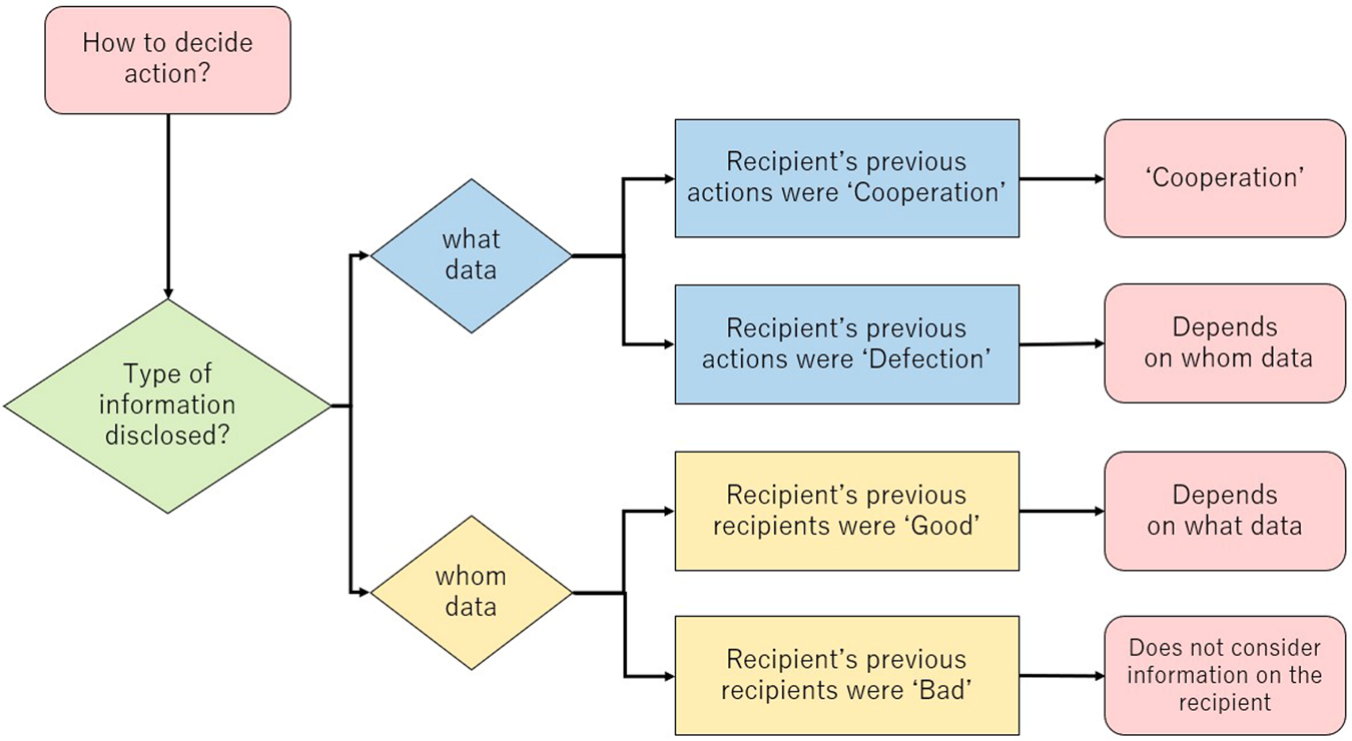
Flowchart of decision-making using information about recipients. This conceptual flowchart represents a summary of the factors determining cooperative behavior in Models 4 to 7 in Table 1, where the labels ‘G’ and ‘B’ correspond to ‘3 or more’ and ‘2 or less’ C actions, respectively, in the previous five rounds. When a subject first receives what data about their recipient, they obtain either ‘C’ or ‘D’ as the recipient’s action. If ‘C’, the subject decides to cooperate with the recipient regardless of the content of the whom data. If ‘D’, the subject then receives the related whom data, in which they obtain either ‘G’ or ‘B’ regarding the recipient’s previous recipient. The subject cooperates with of defects against the recipient if the whom data is ‘G’ or ‘B’, respectively. When the subject first receives whom data about the recipient, they obtain either ‘G’ or ‘B’ regarding the recipient’s previous recipient. If ‘B’, the subject does not use this information to determine their action, but considers other information (decisions they made in the previous two rounds) to decide their action. If ‘G’, the subject receives the related what data. The subject cooperates with or defects against the recipient if the what data is ‘C’ or ‘D’, respectively.

As shown in Fig. 3, subjects’ cooperative actions depend on the content of the information about their recipients. When the recipients’ previous actions were ‘C’, the subjects cooperated with them without considering any other information. In contrast, if the recipients’ previous actions were ‘D’, the subjects tended to check whether their refusal to cooperate was justified. In addition, if the recipients’ previous recipients were ‘G’, the subjects tended to check whether their recipients engaged in prosocial action, i.e., cooperation.

Our results highlight the importance of focusing on the what data or first-order information. Subjects cooperated with those who had previously cooperated. This is known as downward indirect reciprocity^8^. This prosociality (cooperation to cooperation) reflects a chain of prosociality (cooperation to cooperation to cooperation), which is supported by Model 6 in Table 1. Moreover, our results support the proposition that people consider so-called justified defection.

Surprisingly, if the recipients’ previous recipients were ‘B’, the subjects did not consider the information regarding the recipients, and based their decisions on other information. The subjects looked at the information about their recipients but did not consider it. This selective inattention is supported by the Fisher’s exact test values in relation to the information disclosing behaviour shown in Fig. 2c.

Although economic models are usually based on the assumption that agents are unconstrained in their ability to process information, economists have long recognized that individuals have limited cognitive abilities^33^. A series of empirical studies on these limited abilities has repeatedly shown that the human trait of rational inattention^34^ is used as a shortcut for complex information processing. For example, Lactera *et al*.^22^ found left-digit bias (a tendency to focus on the leftmost digit of a number while partially ignoring other digits) in the processing of odometer values after analysing more than 22 million wholesale used-car transactions.

Several studies have observed not only defensive inattention in relation to complex information processing, but also proactive inattention. Cheremukhin *et al*.’s experimental evidence^23^ strongly suggests the conscious disregard of information rather than limitations in a decision-maker’s cognitive abilities. The majority of errors by subjects in their experiment arose from deliberate decisions to ignore some of the available information.

## Discussion

Our study provides support for the existence of proactive inattention when making decisions regarding cooperation. Although the subjects in our experiment willingly received information regarding other players, they made their decisions after ignoring specific information. As shown in Model 7 in Table 1, they did not consider the what data in their decision-making if the whom data were ‘B’, despite the disclosure rate being more than 60%, as shown in Fig. 2c. Comparing this with the data shown in Model 6 in Table 1, the information that whom data were ‘B’ is regarded as a trigger for selective inattention.

We focused on understanding selective inattention when subjects made a decision about an action (i.e., action rules) rather than when they assess an action (i.e., assessment rules). This is in contrast to many previous studies that focus on models aimed at determining the proper way to attribute reputations, i.e., based on the assessment rules in indirect reciprocity. Theorists have explored the use of higher-order information about other players to explore cooperative regimes. Ohtsuki and Iwasa’s exhaustive analysis^4^ considers information up to the third order (i.e., one’s own reputation) while Santos *et al*.’s latest approach^35^ deals with fourth-order information (past information). In contrast to their approaches, we focused on the information that subjects did not use. Humans have limited cognitive abilities, and thus may use simpler information processing rules. Our results in relation to selective inattention enable this new perspective to be further explored.

Okada’s theoretical study of staying^32^ gives a hint of the rationality of selective inattention. Since Nowak and Sigmund’s (1998) study, the theory of indirect reciprocity has been based on the strict application of assessment rules, which has led to the controversy on justified defection. In striking contrast to this, our results provide a new alternative in terms of social justice, overcome the controversy, and suggest the limitations of the strict application of not only rules but also laws (e.g., judicial discretion).

## Methods

### Experimental design details

The experiments were approved by the Research Ethics Committees at Soka University and Rissho University, and were performed in accordance with relevant guidelines and regulation. Participation was by informed consent. The experiment was conducted using ECoSoS, a self-developed computerized system for which the coding is available upon request.

Subjects played more than 50 rounds of an ‘indirect helping game.’ They were not informed of the number of rounds because endgame effects were strictly avoided. Initially, each subject was given 2,000 points and told that their earnings at the completion of the experiment would be cashed out at a rate of 100 points = 40 JPY as compensation for their participation. In each round, the system assigned an anonymous recipient, called Player X, to each subject. Subjects never knew the identity of their recipients. Subjects were given two options, and they signified their choice by clicking on either a red or blue button. The red choice (Cooperation or ‘C’) meant that subjects increased the earnings of their recipient by 300 points and decreased their own by 100 points. The blue choice (Defection or ‘D’) meant that neither the subject’s earnings nor those of their recipient changed. In addition to having a recipient assigned to them, a subject had a virtual donor assigned to them by the system. The donor decided whether ‘C’ (receiving 300 points) or ‘D’ (0 points) was applied to the subject, and subjects were made aware of their donor’s decision after they had made their own decision.

Subjects were assigned donors and recipients by a computer program, and thus never interacted with the other subjects. There were four types of donor: perfect cooperators (10% probability), perfect defectors (10% probability), random behaviour (20% probability), and discriminators (60% probability). The population percentages were determined on the basis of our empirical intuition. Perfect cooperators always chose ‘C’ and perfect defectors always chose ‘D’. Random behaviours made their decisions at random (50–50) regardless of the situation. Discriminators considered their recipients’ last five decisions, and chose ‘C’ only if the majority number of recipients indicated cooperation (in other words, three or more).

A subject can receive at most four pieces of information about their recipient, as shown in Fig. 1 before making their decision (by clicking on either the red or blue button). During the first ten rounds, a subject can reveal any number of pieces of information, and thus the maximum number of items of disclosed information is capped at ten.

### Data collection

A total of 152 students from Soka University (55%) and Rissho University (45%) participated in the experiment. The average age was 20.5 years (standard deviation: 1.22 years) and 64% were male. Almost 100% of the subjects were Japanese. In terms of the area of study, 60% were studying business, 17% in economics, 13% in letters (humanities), 6% in law, and 4% in other fields. The experiment lasted between 60 and 100 minutes. Participants earned on average JPY 3,921 (standard deviation: JPY 1,114, max: JPY 6,100, min: JPY 1,600).

### Statistical analysis

See the statistical analysis in Supplementary Information for details of the model specifications.

## Electronic supplementary material

Supplementary Information

## Data Availability

The data supporting the findings of this study are stored in a Dryad data package titled ‘Data of the ECOSOS project, part 1’ and can be found at https://osf.io/pjfuh/.
