# Supplementary material for: Experimental evidence of selective inattention in reputation-based cooperation
Source: Sci Rep. 2018 Oct 4;8:14813. doi: 10.1038/s41598-018-33147-x (PMC6172233; doi:10.1038/s41598-018-33147-x)
Supplement: Supplementary file 1 — Supplementary Information [file 41598_2018_33147_MOESM1_ESM.pdf]

Supplementary Information for

“Experimental evidence of selective inattention in reputation-based cooperation”

Isamu Okada*, Hitoshi Yamamoto, Yoshiki Sato, Satoshi Uchida, and Tatsuya Sasaki

*Correspondence to: okada@soka.ac.jp

1. Supporting Statistical Analysis

2. Experimental procedure

3. Experimental instructions

1. Supporting Statistical Analysis

Table S1. The number of items of each type of data presented in Fig. 2c in the main text.

	What data were 'C'	*What data were 'D'*
# disclosing whom data	1,518	1,587
# not disclosing whom data	4,005	3,929

	Whom data were 'G'	*Whom data were 'B'*
# disclosing what data	1,333	1,267
# not disclosing what data	635	757

For example, when what data disclosed was 'C', the number of items of whom data that was disclosed was 1,518 for all pairs of all subjects.

Table S2. VIF values of factors presented in Table 1 in the main text.

**Model**	(1)	(2)	(3)	(4)	(5)	(6)	(7)
what data		**1.031**		**1.082**	**1.042**	**1.194**	**1.014**
whom data			**1.030**	**1.072**	**1.043**	**1.025**	**1.012**
recent decision	**1.014**	**1.130**	**1.186**	**1.199**	**1.182**	**1.193**	**1.129**
round	**1.485**	**1.356**	**1.318**	**1.309**	**1.574**	**1.657**	**1.885**
current total earnings	**1.627**	**1.596**	**1.632**	**1.580**	**2.005**	**1.929**	**2.078**
decision received	**1.156**	**1.225**	**1.355**	**1.164**	**1.318**	**1.256**	**1.138**

The variance inflation factor (VIF) is the ratio of the variance in a model with multiple terms to the variance in a model with only one term. VIF values of all factors are sufficiently less than 10, and thus multicollinearity can be ignored.

2. Experimental procedures

We ran seven sessions in the computer laboratories of either Soka University or Rissho University. Once the experiment had begun, the subjects were no longer allowed to communicate with each other. Printed instructions in Japanese (see Subsection 3 for an English translation) were distributed and read out loud by one of the experimenters. To promote the subjects' understanding of the experiment, the experimenter also presented the instructions using PowerPoint slides. Sessions finished with a questionnaire including items about subjects' demographic background (omitted from the paper). Finally, subjects were paid.

3. Experiment Instructions

(1) Setting

During the experiment, you are not allowed to talk to or communicate in any other way with the other participants. Hence, you will be seated apart from each other, not only to prevent communication but also to avoid the possibility of observing other participants' behavior, either intentionally or accidentally. The experiment lasts for about 60 to 90 minutes. It is recommended that you go to the bathroom before starting. You are allowed to take a break during the experiment. Please let one of the staff know if this is necessary.

(2) Your goal

You will be allocated 2,000 points as your initial earnings. Your aim is to gain as many points as possible during the experiment. The payment you receive will be determined by your final points total.

(3) Calculation of payment

At the end of the experiment, you will receive payment consisting of a participation fee and an additional fee. The participation fee is JPY 1,000, which you will be entitled to receive upon providing informed consent. The additional fee will be determined by your final points total. The formula used for calculation is as follows: [The additional fee] = [Your final points] * 0.4, where all amounts are rounded up to the nearest JPY 100. For example, if you had accumulated 4,100 points by the end of the experiment, you would receive JPY 2,700 because 1,000 +4,100 * 0.4 = JPY 2,640, which is rounded up to JPY 2,700. If the final points total is a negative amount, the additional fee is zero.

(4) ID and password

You will access the experiment through a Web browser. To log in to the system, you will be given an ID and a password. The staff never record who has which ID.

(5) Details of the game

The game will involve human subjects and virtual participants created by a computer program. All participants are called 'players'. The experiment consists of multiple rounds. No player will know how many rounds they will play. The game will end suddenly at the end of a round. In each round, your recipient is assigned to you by the computer system. You never know who your

recipient is: instead, the recipient is always called ‘Player X’. You will decide between two options by clicking on either the red or the blue button. The options are as follows:

Red: You will increase the earnings of Player X by 300 points and decrease your own earnings by 100 points.

Blue: Neither player’s earnings will either increase or decrease.

Prior to making your choice, a screen on information disclosure for Player X will be displayed to you. On this screen, you can disclose two types of information: what Player X did, and to whom, in the previous five rounds. The disclosable information is as follows.

Information about who Player X’s recipient was in the nth-last round, where n is an integer between 1 and 5: this is information about the recipient assigned to Player X by the system in the nth-last round. If you click the button for this information, you will see how many times the recipient chose the red button in the five most recent rounds. The number is an integer between 0 and 5.

Information about what Player X chose in the nth-last round: this is information about Player X’s choice for the recipient assigned to Player X by the system in the nth-last round. If you click the button for this information, either a red or a blue symbol is displayed.

In each round, you can disclose at most four pieces of information without cost, and thus you should consider which information will be disclosed. During the first ten rounds, you can disclose any amount of information, and thus you should try to disclose as much information as possible in the first ten rounds. Note that the information disclosure screen has two versions, which will be displayed in alternate rounds. If you no longer wish to see the information disclosure screen, press the ‘Finish disclosure’ button. You can move to the next screen by choosing one of two options (clicking the red or the blue button). Any information you disclosed on the information disclosure screen will disappear, and thus you should only move to this screen after you have made your choice.

This is the end of a round. As well as assigning Player X, the system also assigns a donor to you. The assigned donor also has a choice of two options, and you will learn the donor’s decision (by

clicking the red or the blue button and updating your earnings) at the end of each round. The donor makes their choice after referring to information about you, just as you did for your recipient. Therefore, you should make your choices carefully because they may influence the choices made by your future donors. Additionally, the first few rounds of the experiment have insufficient information on the past, and thus the system will provide past information randomly.

(6) Process after the game

When the game has finished, you should wait quietly for a staff member to give you a questionnaire and a receipt. When you have completed the questionnaire, give the form to a staff member and collect your payment. After receiving your payment, you should quietly leave the room.
